# Improved Wallis Dodging Algorithm for Large-Scale Super-Resolution Reconstruction Remote Sensing Images

**DOI:** 10.3390/s17030623

**Published:** 2017-03-18

**Authors:** Chong Fan, Xushuai Chen, Lei Zhong, Min Zhou, Yun Shi, Yulin Duan

**Affiliations:** 1 School of Geoscience & Info-Physics, Central South University, Changsha 410083, China; fanchong@csu.edu.cn (C.F.); leizhong1030@gmail.com (L.Z.); ryumin106@gmail.com (M.Z.); 2 State Key Laboratory of Information Engineering in Surveying, Mapping and Remote Sensing, Wuhan University, Wuhan 430079, China; 3 Geomatics Engineering Investigation of China Gezhouba Group Co., Ltd., Yichang 443000, China; 4 Institute of Agricultural Resources and Regional Planning, Chinese Academy of Agricultural Sciences, Beijing 100083, China; shiyun@caas.cn

**Keywords:** super-resolution reconstruction, large-scale image, Wallis dodging, overlapping region, optimal seam line, seam elimination

## Abstract

A sub-block algorithm is usually applied in the super-resolution (SR) reconstruction of images because of limitations in computer memory. However, the sub-block SR images can hardly achieve a seamless image mosaicking because of the uneven distribution of brightness and contrast among these sub-blocks. An effectively improved weighted Wallis dodging algorithm is proposed, aiming at the characteristic that SR reconstructed images are gray images with the same size and overlapping region. This algorithm can achieve consistency of image brightness and contrast. Meanwhile, a weighted adjustment sequence is presented to avoid the spatial propagation and accumulation of errors and the loss of image information caused by excessive computation. A seam line elimination method can share the partial dislocation in the seam line to the entire overlapping region with a smooth transition effect. Subsequently, the improved method is employed to remove the uneven illumination for 900 SR reconstructed images of ZY-3. Then, the overlapping image mosaic method is adopted to accomplish a seamless image mosaic based on the optimal seam line.

## 1. Introduction

Remote sensing (RS) images are increasingly being used in agriculture, mainly in the monitoring of crops, resources, and disasters. In particular, high-resolution RS images play a significant role in agriculture, aiding with the development of agricultural production and research into the fine and quantitative stage. However, high-resolution images are generally quite expensive. Super-resolution (SR) image reconstruction is a technique that recovers a high-resolution image from several low-resolution images using the non-redundant information among them [1,2]. A sub-block algorithm is usually used in the SR reconstruction of images to improve the operation efficiency and reduce the consumption of computer memory. The continuity and correlation between blocks are ignored, particularly at the block junctions. The gray-level distribution will always be discontinuous, resulting in inconsistent color of the large-scale mosaic image. In other words, the brightness and contrast distribution of images are uneven and an obvious seam line is observed [3]. Therefore, the color differences among images should be diminished before image mosaicking.

The uneven illumination phenomenon of RS images, which directly affects the quality of images and leads the mosaic result to exhibit obvious differences in luminance or color [4], is a significant problem in image processing. To date, two kinds of dodging processing algorithms exist, depending on the number of images involved. Mask dodging [5,6], retinex uniform light [7,8], and homomorphic filtering dodging [9,10] ensure color balance inside a single image, whereas histogram matching, dodging based on information entropy, and Wallis filter-based dodging deal with the color difference between images. Histogram matching [11,12,13,14] is able to reduce the difference in brightness and hue among image objects by correcting the shape of the histogram. However, this method adjusts the mean and variance to fit both the reference and target images by directly changing the histogram shape. Different internal features of images reflect the differences in the histogram shape of the image. Thus, if the difference is large, histogram matching may change the relative distance between gray levels, leading to color deviation of an image with different internal features. The dodging method based on information entropy eliminates or weakens the color difference through entropy mapping, whose theory is that two images have the same amount of information in the overlapping region. The Wallis filter is usually used to adjust the gray mean and variance of images to a given level, as its effectiveness and many improvements are put forward by researchers. Zhang Li investigated the principles and characteristics of the Wallis filter and determined that the Wallis filter, which has a local adaptive function, can enhance the contrast of images while suppressing noise [15]. On this basis, Li De-ren proposed a dodging approach for multiple images with the Wallis filter by adjusting the mean and variance of the image, which relies on the linear transfer relationship and causes color deviation of images that are far from the reference image [6]. Aiming at the characteristics of a large-scale seamless image database, an improved color balance method, which considers local and global information according to the Wallis adaptive filter, was proposed by Wang Mi. Not only can this method solve the color balance problem effectively, but it can also preserve radiometric resolution easily [16]. Zhang Deng-rong employed a whole-image color adjustment based on the Wallis transformation in the color adjustment step, and separately adopted a weighted smoothing method based on the optimal seam line and a compulsion correction method according to the size of the overlap area [17]. Zhou Li-ya presented a single image based on the Wallis filter-based dodging algorithm, which achieved single-image dodging through reasonable blocking and automatically achieving the statistics of the mean and variance [18]. Wang Ye and Zhang Han-song selected the Wallis filter parameters using the optimal index factor, and improved the Wallis dodging algorithm based on the moment matching balancing algorithm [19]. Chen Ke obtained large-scale seamless DOM (digital orthophoto map) data and disposed of the problem of consistency by using two algorithms. One is the dodging method for a single image based on the Gaussian filter, the other is the uniform color for multiple images based on the Wallis filter [20]. Luo Si proposed an improved dodging algorithm based on the Wallis principle, which can achieve illumination consistency and contrast effectively [21]. Tian Jin-yan developed a seam elimination approach for unmanned aerial vehicle images based on Wallis dodging, which was introduced to adjust the difference of brightness between the matched images and Gaussian distance weight enhancement that can fuse the two matched images in the overlapping region [22].

The existing Wallis dodging algorithm is only suitable for keeping the color and brightness consistency between two images or a few images, whose parameters usually depend on the selected standard image from the original images, which has many different criteria. However, for large format images, the uneven distribution of brightness and contrast cannot be removed completely because of the propagation of errors. In view of the aforementioned problems, this study proposes an innovative color adjustment and smoothness transition method, which successfully achieves color consistency processing and a seamless image mosaic. The improved Wallis dodging algorithm mainly has two improvements:

(1) A weighted method is used to calculate the Wallis filter parameters for each image.

(2) A new method of weighted iteration of adjacent images is proposed to eliminate the spatial transfer error caused during large-scale image adjustment.

## 2. Methodology

### 2.1. Super-Resolution Reconstruction

Image SR reconstruction, which was first proposed by Harris and Goodman in the 1960s, is the process of producing one or a series of high-resolution image(s) by exploiting hidden information from one or a sequence of low-resolution original image(s). Generally, SR methods can be divided into two categories, namely, frequency domain method and spatial domain method [23,24]. A classical approach of minimizing a regularized energy function, which is invulnerable to noise, is adopted in this study [25]. The complete energy function with four terms takes the following form:

(1) $$E\left(f,h\right)=\overset{K}{\underset{k=1}{\sum }}{\left|\left|{\mathrm{DH}}_{k}f-g_{k}\right|\right|}^{2}+\alpha Q\left(f\right)+\beta_{1}R\left(h\right)+\beta_{2}Q\left(h\right)$$

The first term measures fidelity to the data. The remaining three terms, i.e., α, $\beta_{1}$, and $\beta_{2}$, are regularization terms with positive weighting constants that attract the minimum of E to an admissible set of solutions. Q(f) is a smoothing term used to suppress noise in low-resolution images. The point spread function regularization term R(h) directly follows from the conclusions of the previous section. Q(h) assumes that the fuzzy function is a smooth variation. 

### 2.2. Wallis Dodging

The Wallis filter is actually a local image transform, which ensures that the standard deviations and means of the image at different locations are approximately equal. This phenomenon produces good local contrast throughout the image, while reducing the overall contrast between bright and dark areas. The small amount of gray information of the image is enhanced [18], which ensures that the Wallis filter has a special effect on images with low contrast.

The Wallis dodging algorithm can be used to modify the brightness and contrast of multiple images. According to the statistical mean and variance of the selected reference image, the linear distribution of the gray level of the target image is adjusted by the Wallis filter using Equation (2):

(2) $$f\left(x,y\right)=\left(k\left(x,y\right)-m_{k}\right)\alpha +\beta$$

where k(x,y) is the gray value of the target image, f(x,y) is the gray value of the results of Wallis dodging, and α and β are multiplicative and additive coefficients, which can be calculated using Equation (3):

(3) $$\{\begin{array}{c} \alpha =\frac{{c\;s}_{f}}{{c\;s}_{k}+\left(1-c\right)s_{f}}\\ \beta =bm_{f}+\left(1-b\right)m_{k} \end{array}$$

where $m_{k}$ and $s_{k}$ are the mean and standard deviation of the target image, $m_{f}$ and $s_{f}$ are the mean and standard deviation of the reference image, *c* ∈ [0,1] is the expansion coefficient for the variance value, and *b* ∈ [0,1] is the luminance coefficient for the mean value. The mean of the image is close to $m_{f}$ when *b* tends to 1, whereas the mean is close to $m_{k}$ when *b* tends to 0. The equation of the classical Wallis transform is expressed in Equation (4), where the parameters (*b* and *c*) are equal to 1:

(4) $$f\left(x,y\right)=\left(k\left(x,y\right)-m_{k}\right)\times \frac{s_{f}}{s_{k}}+m_{f}$$

**Figure 1:**
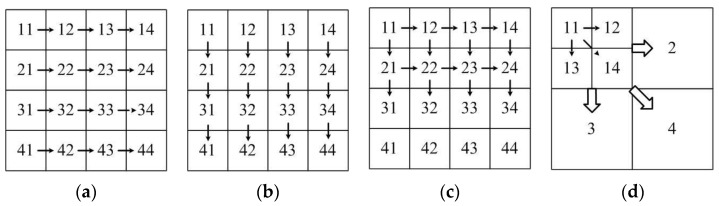
Schematic diagram of the sequence of image adjustment: (**a**) left image benchmark; (**b**) above image benchmark; (**c**) left image cross; and (**d**) quadtree.

In general, Wallis dodging involves two images (i.e., the reference and target images). When several images are obtained, the images should be dealt with individually. Four processing sequences are mainly used to adjust the color of multiple images, as shown in Figure 1. Each image is adjusted based on its adjacent left or upper image, as shown in Figure 1a,b. Another method is shown in Figure 1c. The images, except those located on the boundary of the block images, are adjusted twice. An adjustment method based on the four-fork tree is proposed according to the idea of quadtree coding [26]. Four adjacent images are defined as child nodes. Four child nodes form the parent node. Then, four parent nodes are calculated to complete the entire image processing. The correlation between adjacent images is considered in this method, and the suture transition processing method [27,28] is used for color images. Although the processing results of the method are unaffected by the processing order [29], this method is limited by the number of images. The small block may lose a considerable amount of gray-level information because of multiple computations.

### 2.3. Seam Elimination

Seam lines are often apparent between parts when RS images are combined in image mosaicking. The seam lines are caused by gray-level differences because of the different conditions under which the parts were recorded. In this study, slight differences are still observed among the tones in the overlapping region of the adjacent images after the Wallis dodging processing. As a result, an obvious stitching line of the image mosaicking result, which reduces the visual quality and application value of the image, is observed. Thus, seam line removal is indispensable to ensure a smooth gray-level transition in the overlapping region of two adjacent images. Two categories of seam line removal methods are available: one is based on wavelet transform [30,31] and the other is based on overlapping image [32]. The theory of the seam line removal method based on wavelet transform is rigorous. However, achieving this method is difficult and the requirement of computer memory is high. The seam line removal method based on overlapping image is simple and widely used to achieve smooth transition in the overlapping region. In this method, the gray value of each pixel at the two sides of the seam line is corrected according to the gray difference between two pixels at the same position at a certain extent of the overlapping areas, as shown in Equation (5). The closer the pixel is to the seam line, the larger the correction value is.

(5) $$I_{i}=IA_{i}+(IB_{i}-IA_{i})\times K_{i}\;K_{i}=\frac{i}{W}\;0≦i≦W-1$$

where $I_{i}$ denotes the gray value of the pixel after processing; $IA_{i}$ and $IB_{i}$ are the pixel gray values of the two-scene image mosaicking in the overlapping area; *W* is the gray smooth width, which is less than the width of the image overlapping area; and *K* is the weight, which shows a linear inverse variation in the smooth range.

The location of the seam line critically influences how effectively any two data sets can be merged. The bisector of the overlapping area can be used as the seam line. However, its effect is not ideal. Thus, searching for a curve as the optimal seam line in the overlapping area, where two adjacent images have the minimum difference, is usually necessary. Many optimal seam line searching methods have been developed in the literature, including Dijkstra’s algorithm-based methods [33,34,35], dynamic programming-based methods [36,37,38], and graph-cuts-based methods [39,40]. Ancillary data is also applied to detect the seam line in some algorithms, such as digital surface model, vector roads, building map, and lidar point clouds [41,42,43]. In this study, a minimum connected path is sought by certain criteria based on the difference of images. This path is created by the differences of the gray values in the overlapping area.

**Figure 2:**
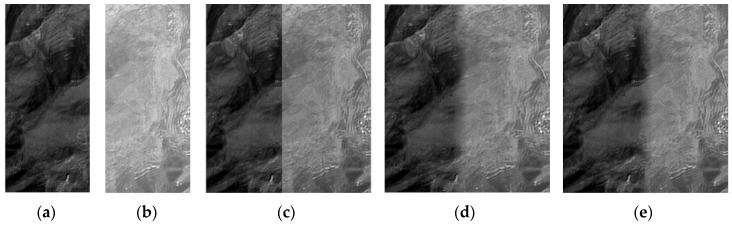
Image mosaicking results: (**a**) original left image; (**b**) original right image; (**c**) image mosaicked directly; (**d**) image mosaicked with the bisector of the overlapping region as the seam line; and (**e**) image mosaicked with the optimal seam line.

An experiment with two images which exhibit significant gray-level difference, as shown in Figure 2a,b, is conducted to verify the effect of the stitching algorithm. The gray-level difference of the mosaicking position is relatively significant, such that an obvious seam line in the direct image mosaicking exists, as shown in Figure 2c. The result shown in Figure 2d is spliced at the bisector of the overlapping region, in which the seam line is not eliminated completely. The result of removing the seam line based on the overlapping area with an optimal seam line is shown in Figure 2e. The smooth transition is natural in panorama, and a perfect visual effect is achieved.

## 3. Improved Wallis Dodging

**Figure 3:**
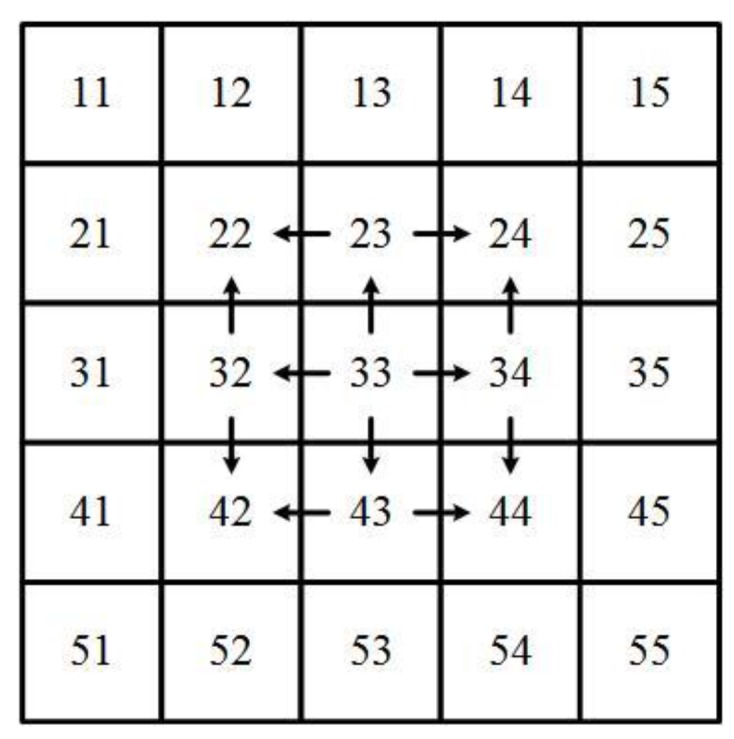
Weighted adjustment sequence.

The existing Wallis dodging algorithm is only suitable for keeping the color and brightness consistency between two images or a few images. The adjustment order and parameter calculation methods are improved to ensure that the Wallis dodging method is suitable for color balance of large-scale SR images. The processing procedure of the proposed algorithm is that the image in the middle is selected as the first standard image and other images in four directions are adjusted gradually, as shown in Figure 3. The images at the same line or column as the first image are modified based on a contiguous image. The other images are based on its two adjacent images.

**Figure 4:**
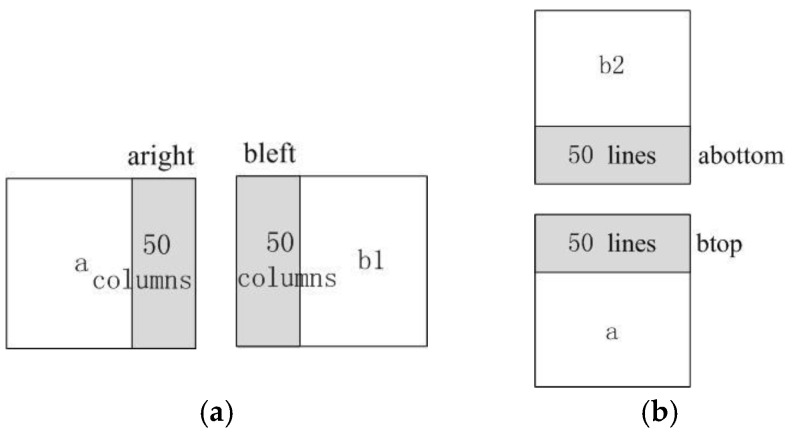
Parameter calculation: (**a**) left and right overlapping diagram and (**b**) upper and lower overlapping diagram.

The traditional Wallis dodging algorithm generally involves a reference image and a target image. However, the target image may be adjusted according to two target images in the proposed method. In this study, two adjacent images are defined as a and b. Their overlapping areas in the horizontal direction are a right and b left, as shown in Figure 4a, whereas their overlapping areas in the vertical direction are a bottom and b top, as shown in Figure 4b. If the image to be processed and the first standard image are at the same line or column, the parameters $m_{k}$, $m_{f}$, $s_{k}$, and $s_{f}$ are calculated according to the overlapping region of the target image and its adjacent image. Otherwise, two abutting images should be considered. For example, image a and right image b1 below image b2 are contiguous, as shown in Figure 4. The means $m_{k1}$, $m_{f1}$, $m_{k2}$, and $m_{f2}$ and the variances $S_{k1}$, $S_{f1}$, $S_{k2}$, and $S_{f2}$ are computed based on the overlapping area of three images. Then, the parameters are formulated, as expressed in Equation (6):

(6) $$m_{k}=P_{1}\times m_{k1}+P_{2}\times m_{k2}\;m_{f}=P_{1}\times m_{f1}+P_{2}\times m_{f2}\;s_{k}=P_{1}\times S_{k1}+P_{2}\times S_{k2}\;s_{f}=P_{1}\times S_{f1}+P_{2}\times S_{f2}$$

where $P_{1}$ and $P_{2}$ denote the weighting coefficients, which are related to the difference of the means of the target and reference images. The purpose of the Wallis filter is to adjust the mean of the target image to the level of the reference image. The difference reflects the degree of adjustment. Accordingly, the parameters are calculated using Equation (7) to ensure that the gray values of the adjusted image are closest to the value of the adjacent two images:

(7) $$P_{i}=\frac{\mathrm{ABS}\left(m_{ki}-m_{fi}\right)}{\mathrm{ABS}\left(m_{k1}-m_{f1}\right)+\mathrm{ABS}\left(m_{k2}-m_{f2}\right)}\;\left(i=1,\;2\right)$$

## 4. Experimental Results and Discussion

ZY-3 is the first civilian high-resolution optical transmission mapping satellite in China, which is equipped with four sets of optical cameras, including a panchromatic TDI CCD camera with a ground resolution of 2.1 m, two sets of panchromatic TDI CCD cameras with a resolution of 3.6 m, and a multispectral camera with a ground resolution of 5.8 m. ZY-3 images with a resolution of 3.6 m are divided into sub-blocks to verify the validity of the proposed algorithm. Then, SR reconstructed images with a resolution of 1.8 m are obtained by the blind SR reconstruction algorithm. The size of these images, which are overlapped 50 pixels, is 1024 × 1024 pixels. The traditional and improved Wallis dodging methods are applied to process these images. All the experiments are realized with MATLAB in this study. The results of the experiments are analyzed in this section.

### 4.1. Image SR Reconstruction

**Figure 5:**
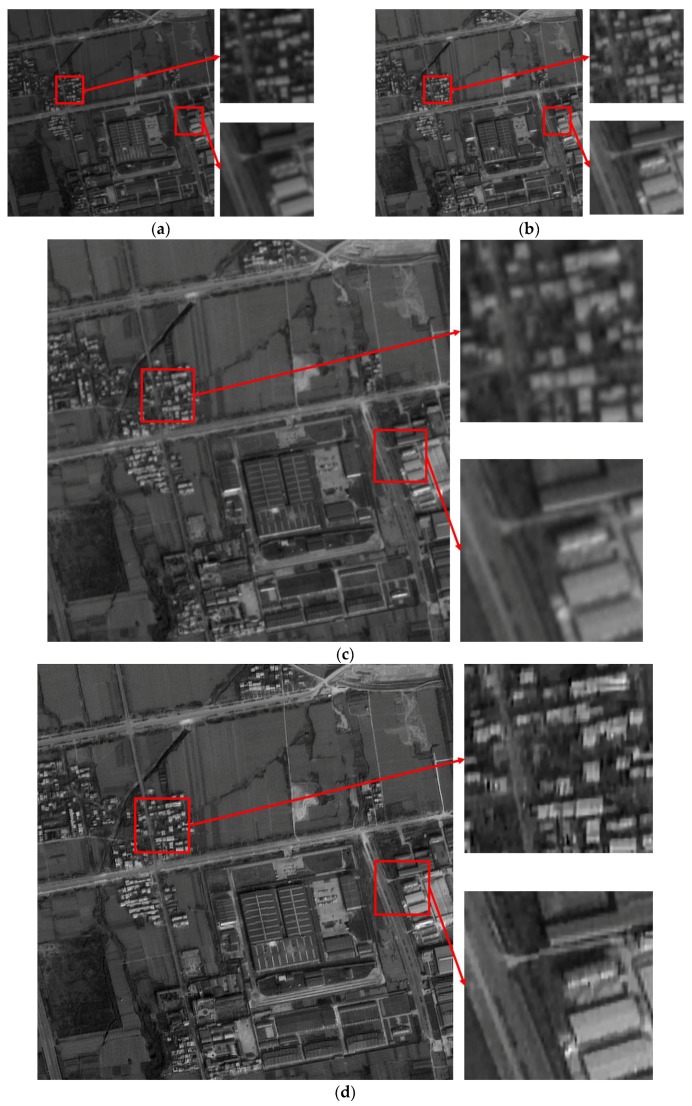
Results of the experiments using two methods: (**a**) original forward image; (**b**) original backward image; (**c**) image based on the bicubic interpolation algorithm; (**d**) image based on the blind SR reconstruction algorithm.

In order to prove the effect and significance of SR reconstruction algorithm, ZY-3 forward and backward panchromatic images with a resolution of 3.6 m, as shown in Figure 5a,b, are reconstructed by the blind SR reconstruction algorithm. The reconstructed image and its details are shown in Figure 5d. The edges of the building and road in the reconstructed image are much clearer and more distinct than that in the original images. Moreover, the result is also better than the image shown in Figure 5c, which is obtained by the bicubic interpolation method. The nominal values of metric Q of two reconstructed images are shown in Table 1. The metric Q of the image based on the blind SR reconstruction algorithm is high, showing that the blind SR reconstruction algorithm has a good visual performance and detail preservation. In summary, Image SR reconstruction can effectively produce robust and clear high-resolution images.

### 4.2. Experiments of Different Dodging Algorithms

**Figure 6:**
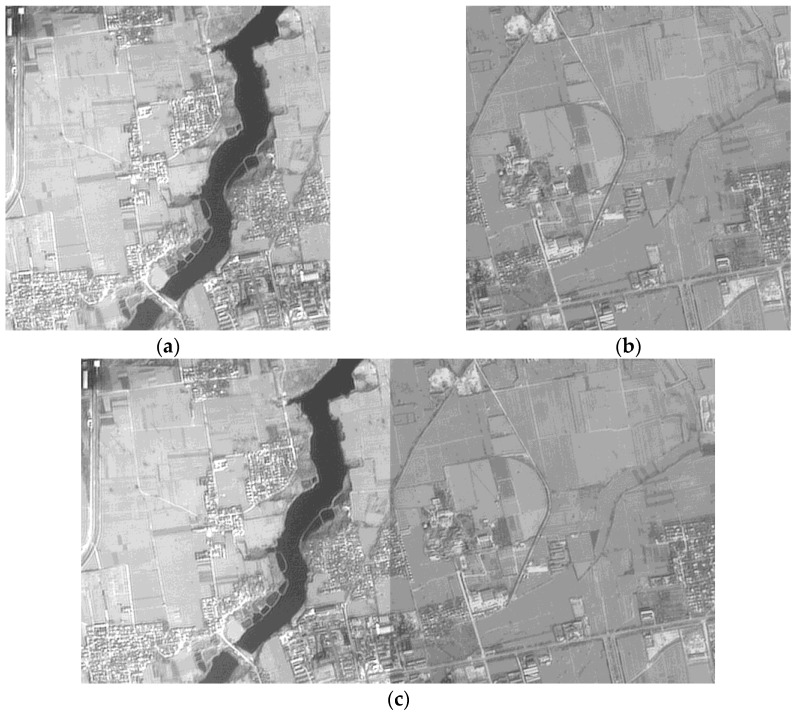

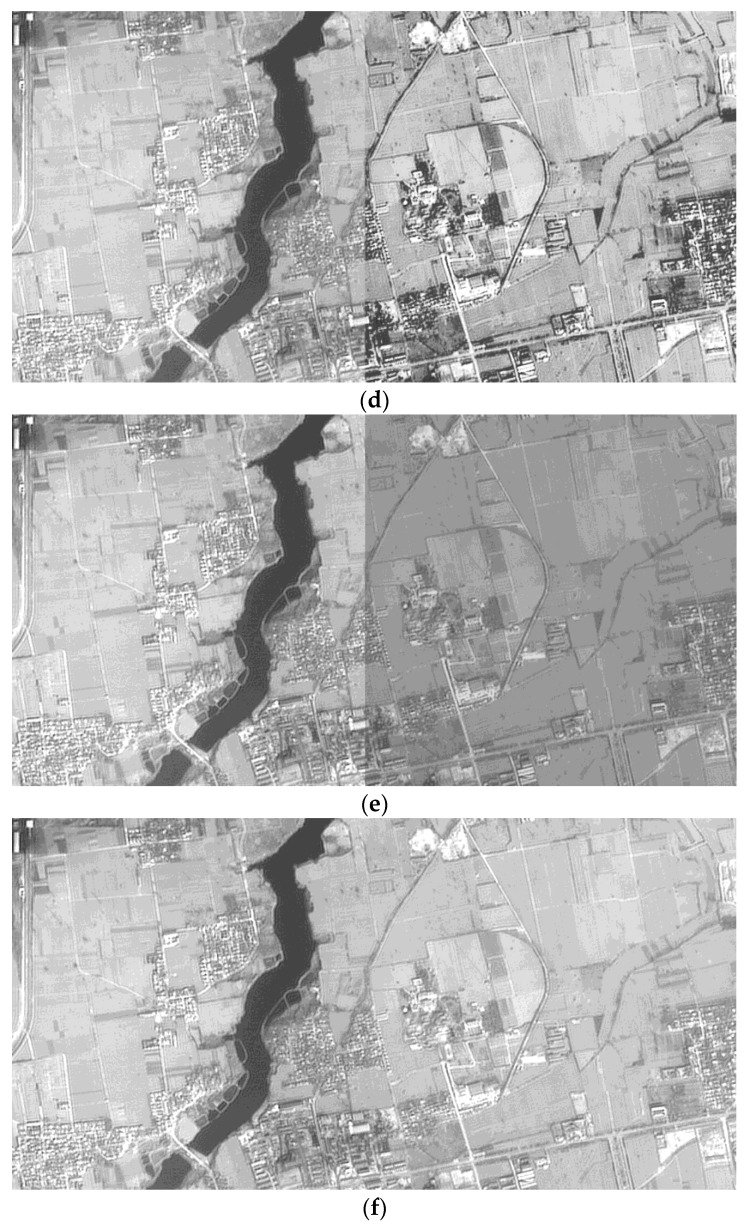
Comparison of different dodging algorithms: (**a**) original left image; (**b**) original right image; (**c**) image mosaicking of original images; (**d**) image mosaicking of two images after dodging based on histogram matching; (**e**) image mosaicking of two images after dodging based on information entropy; (**f**) image mosaicking of two images after dodging based on the Wallis filter.

All the results of different dodging algorithms are affected by the processing sequence. Therefore, two images with large contrast are processed to verify the effect of different dodging algorithms, as shown in Figure 6a,b. An obvious seam line in the image mosaicking without any dodging processing can be observed, as shown in Figure 6c. Although the method based on histogram matching weakens the difference of the original images in brightness and contrast, the seam line in the image mosaicking can also be easily observed, as shown in Figure 6d. Furthermore, the relative distance between gray levels in the processed right image is changed, resulting in the color deviation of the image with different internal features. The effect of the method based on information entropy is worse than that based on histogram matching. The seam line in the image mosaicking shown in Figure 6e almost does not achieve any improvement. The effect of the Wallis dodging method is the best, and the image mosaicking achieves consistent color without a visible seam line, as shown in Figure 6f. In summary, the results shown in Figure 6 indicate that the Wallis filter has the strongest ability to achieve consistency of image brightness and contrast.

In addition, average gradient, information entropy and peak signal-to-noise ratio (PSNR) are considered to estimate the effect of different dodging algorithms on image quality. Average gradient, which reflects the ability of image detail contrast expression, is the image definition. Information entropy represents the amount of image information. PSNR is usually used as an image quality metric for evaluating image processing algorithms. A high PSNR value provides a high image quality. At the other end of the scale, a small value of the PSNR implies high numerical differences between images. From the perspective of image quality assessment indicators, the details of the performance comparisons of the three methods are shown in Table 2. The average gradient, information entropy and PSNR of the image after dodging based on the Wallis filter are highest, which indicates that the Wallis dodging method gives the most abundant amount of information and the highest definition. Hence, the Wallis dodging method is the most widely used method for dealing with the color differences between images.

### 4.3. Comparative Experiment on 64 Images

**Figure 7:**
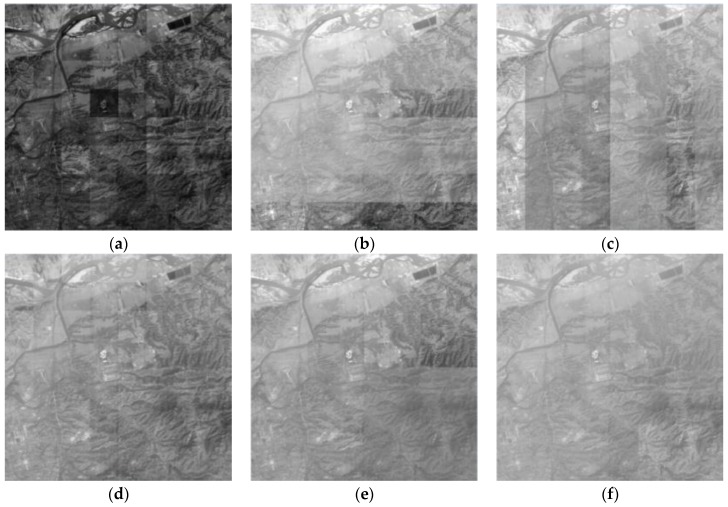
Results of different methods: (**a**) original image; (**b**) result based on left image benchmark; (**c**) result based on above image benchmark; (**d**) result based on left image cross; (**e**) result based on quadtree; and (**f**) result based on the proposed method.

**Figure 8:**
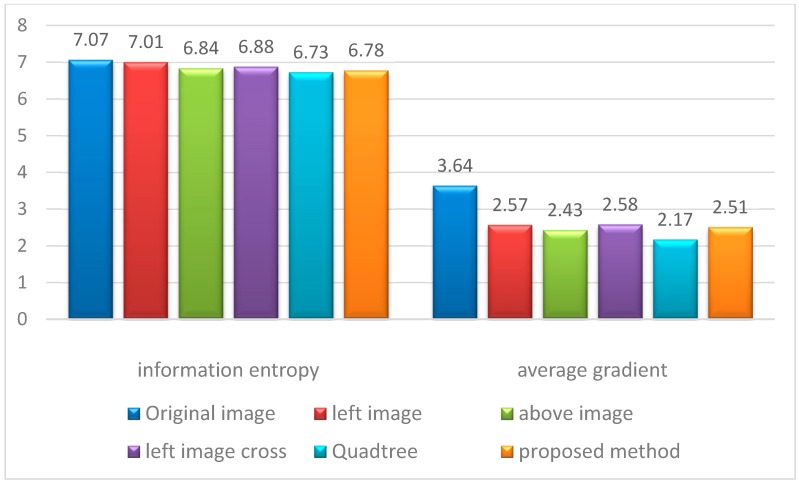
Average gradient and information entropy of the processed results of different methods.

A total of 64 images with large contrast were selected for the comparative experiment and analysis to assess the effect of the proposed algorithm on image quality. Average gradient and information entropy are considered to estimate the specific methods of image dodging. The results of different methods are shown in Figure 7. The details of the performance comparisons of the five methods from the perspective of image quality assessment indicators are shown in Figure 8. The SR image exhibits the most abundant amount of information and the highest definition because of radiation distortion. However, the sub-blocks exhibit the largest difference in brightness and contrast. Thus, the image mosaicking in Figure 7a exhibits a poor visual effect with obvious seam lines. All these dodging methods decrease the average gradient of the image. The left image cross method is the best, whereas the quadtree method is the worst, and the proposed method is at a moderate level. Image dodging generally reduces the amount of image information according to the statistical results of information entropy. The result based on the left image benchmark shows the most abundant amount of information, and the proposed method follows. The result based on the quadtree method has the most loss of image information because of excessive computation.

**Figure 9:**
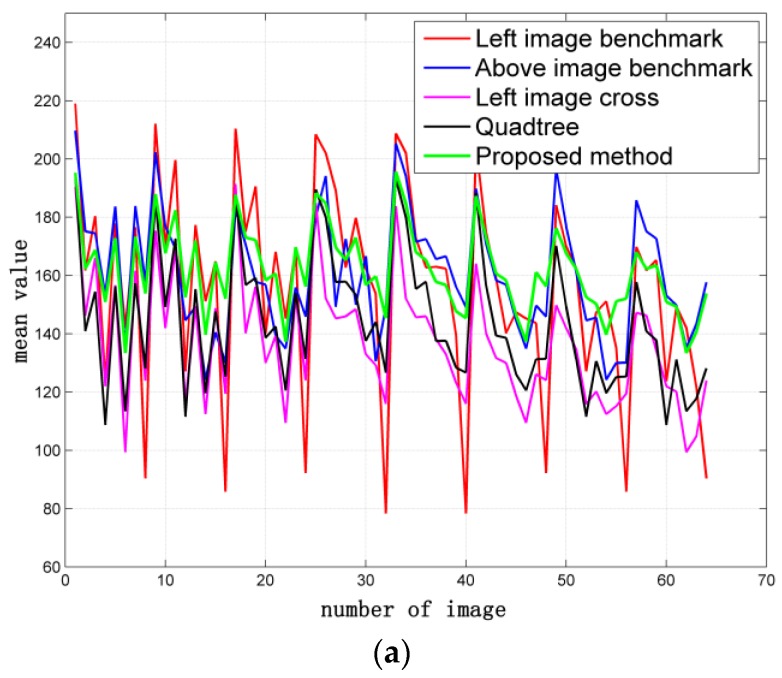

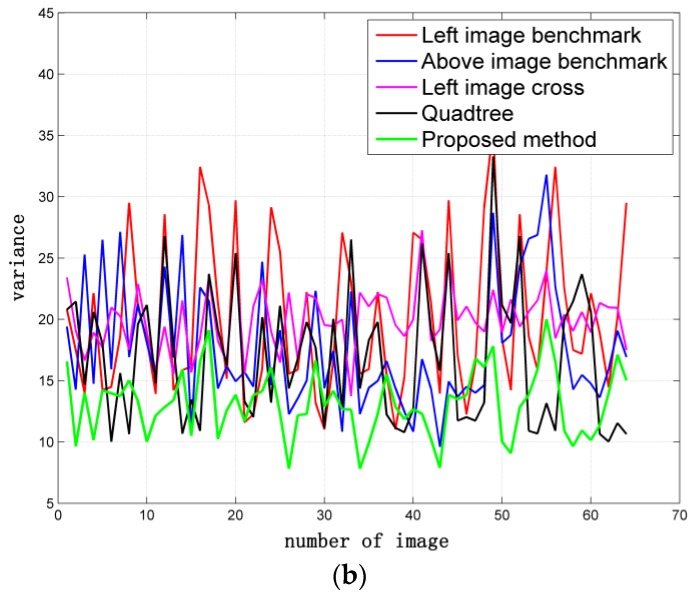
Distribution curve of the mean value and standard deviation of each sub-block image: (**a**) distribution curve of the mean value of each sub-block image; (**b**) distribution curve of the standard deviation of each sub-block image.

The mean value and standard deviation of each sub-block image processed by different dodging methods are computed. The distribution maps are also protracted to estimate the effectiveness of the improved Wallis dodging algorithm, as shown in Figure 9. The distribution curve of the left image benchmark method changes most dramatically and the quadtree method follows. The distribution curve of the left or above image benchmark method is periodic. The variation trend of the distribution curve of the proposed method is relatively stable. This phenomenon indicates that the improved weighted Wallis algorithm is suitable for the color balance of large-scale SR reconstructed images.

### 4.4. Experiment on 900 Images

**Figure 10:**
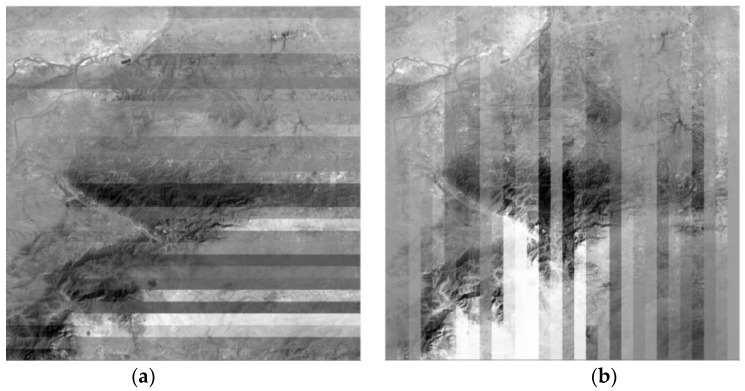

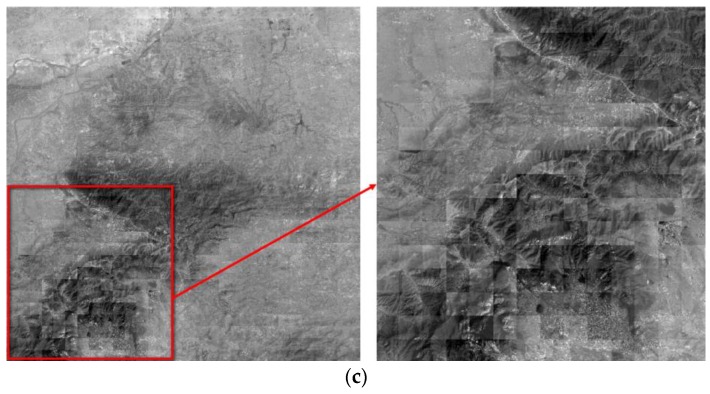
Results based on different sequences: (**a**) result based on left image benchmark; (**b**) result based on above image benchmark; and (**c**) result based on left image cross.

A total of 900 images are processed with the five methods for another comparative experiment to further illustrate the effectiveness of the improved Wallis dodging algorithm for large-scale SR images. The processing methods shown in Figure 1a,b, which ignore the correlation between different rows (columns), are simply suitable for the color balance of a few images. These methods will lead to a belt-form image for large-scale images, as shown in Figure 10a,b. In areas with a relatively large difference of object reflectivity, the phenomenon of exposure will occur, resulting in serious distortion of the image. The processing methods shown in Figure 1c, in which the reference images of one target image are its left and upper adjacent images, may cause partial loss of gray-level information. This phenomenon may also make the contrast of adjacent images obvious, as shown in Figure 10c.

**Figure 11:**
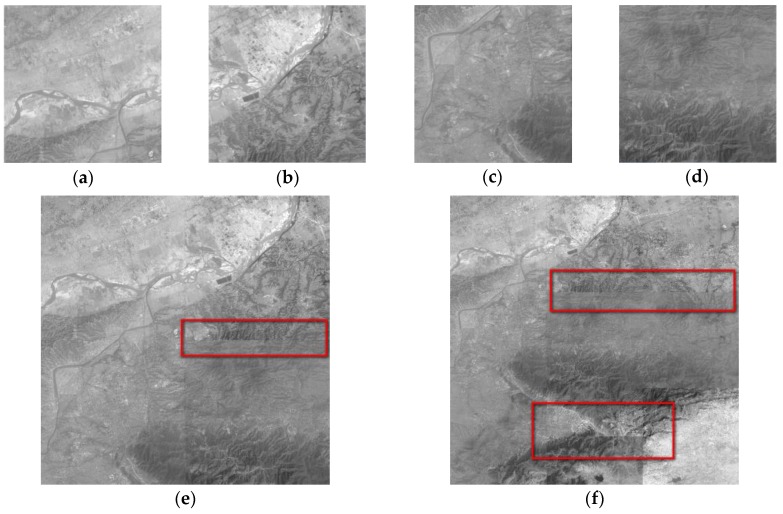
Results of the quadtree method: (**a**) 64 images; (**b**) 64 images; (**c**) 64 images; (**d**) 64 images; (**e**) 256 images; and (**f**) 576 images.

In order to reduce the spatial transfer and accumulation of error, the small sub-blocks are adjusted and then each of the four adjacent blocks are adjusted as a block in the method based on the four-fork tree, as shown in Figure 1d. The consistency of image brightness and contrast can be well achieved for 64 images, as shown in Figure 11a,d. Block effect and loss of gray-level information are observed with the increase in the number of images, as shown in Figure 11e,f.

**Figure 12:**
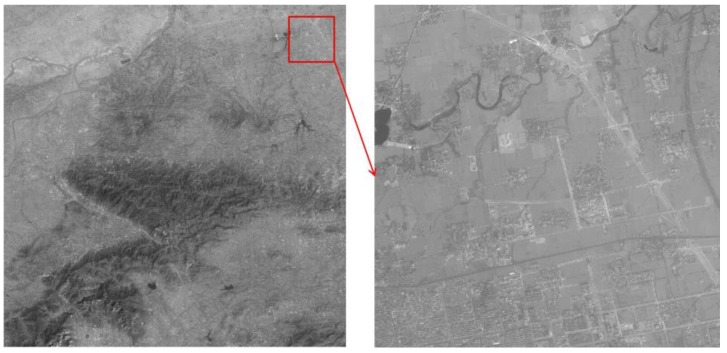
The result and its local amplification effect of the images after image dodging by the improved method.

**Figure 13:**
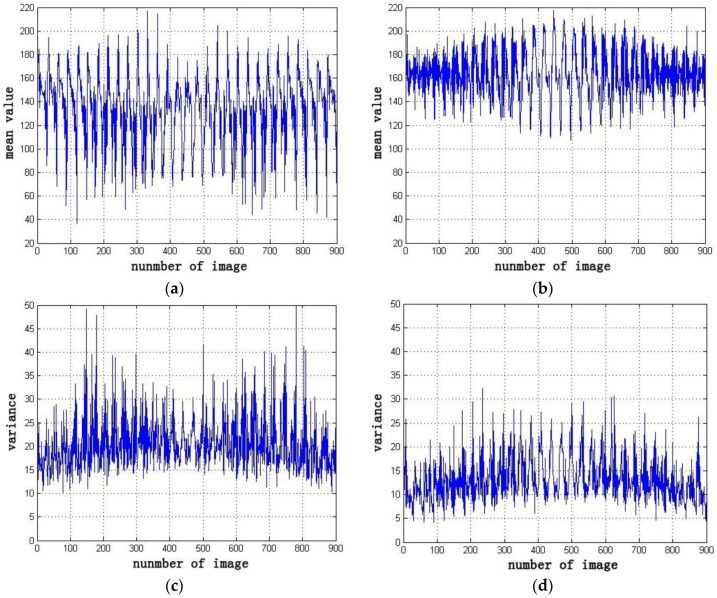
Distribution curve of the mean and standard deviation of the original images and those processed using the Wallis dodging method: (**a**) mean of the original images; (**b**) mean of the images processed using the Wallis dodging method; (**c**) standard deviation of the original images; and (**d**) standard deviation of the images processed using the Wallis dodging method.

The overall color of the original image is dark. The phenomenon of non-uniform brightness and contrast is serious. After image dodging by the improved weighted Wallis dodging method, the images exhibit a consistent brightness and contrast, as shown in Figure 12. The mean value and standard deviation of the original images and processed images using the Wallis dodging method are inputted to the statistical analysis to evaluate the effectiveness of the improved Wallis dodging algorithm quantitatively. The distribution curves are plotted in Figure 13. The mean value and standard deviation of the original images are scattered and changed dramatically, as shown in Figure 13. By contrast, the mean value and standard deviation of the images processed using the Wallis dodging method change more slowly. The change interval is low. This result proves that the proposed approach exhibits the ability of brightness and contrast homogenization.

**Figure 14:**
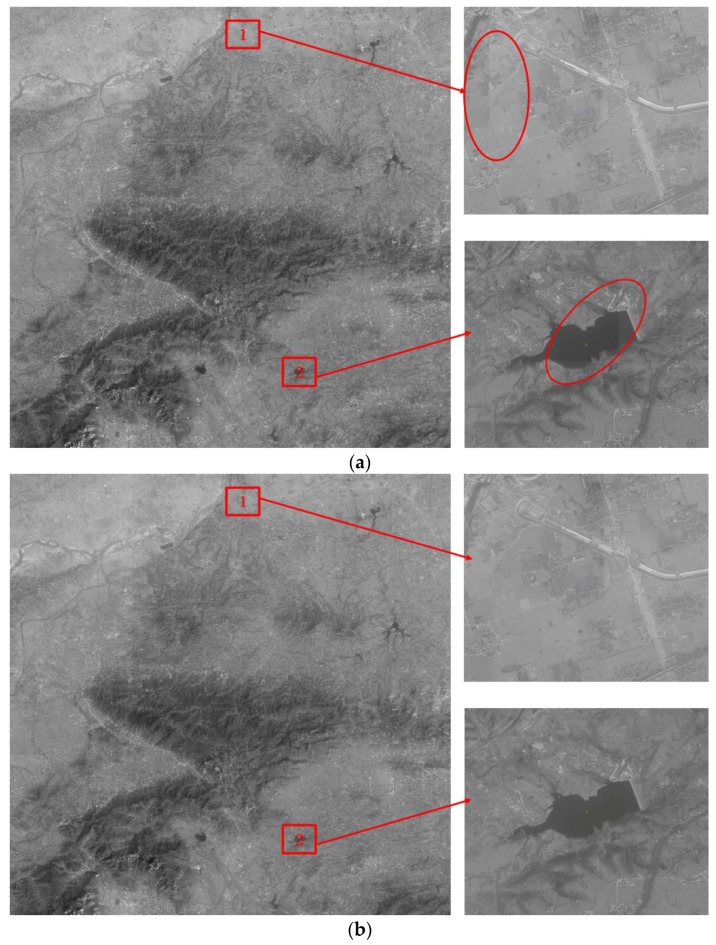
Final results: (**a**) mosaicking result without seam elimination and its local amplification effect on the images after image dodging by the improved method and (**b**) final mosaicking result with seam elimination and its local amplification effect of the images after image dodging by the improved method.

After image dodging by the improved weighted Wallis dodging method, the brightness and contrast of the images are basically the same or close together. The image mosaicking shown in Figure 12 exhibits a good visual effect. However, slight differences are still observed among the tones in the sectional overlapping region of the adjacent images. This phenomenon leads to seam lines, as shown in Figure 14a. Finally, the smooth transition of adjacent images is well achieved by the seam elimination method, which can share the partial dislocation in the seam line to the entire overlapping region. A homogeneous image exhibiting a perfect visual effect is obtained successfully, as shown in Figure 14b.

**Figure 15:**
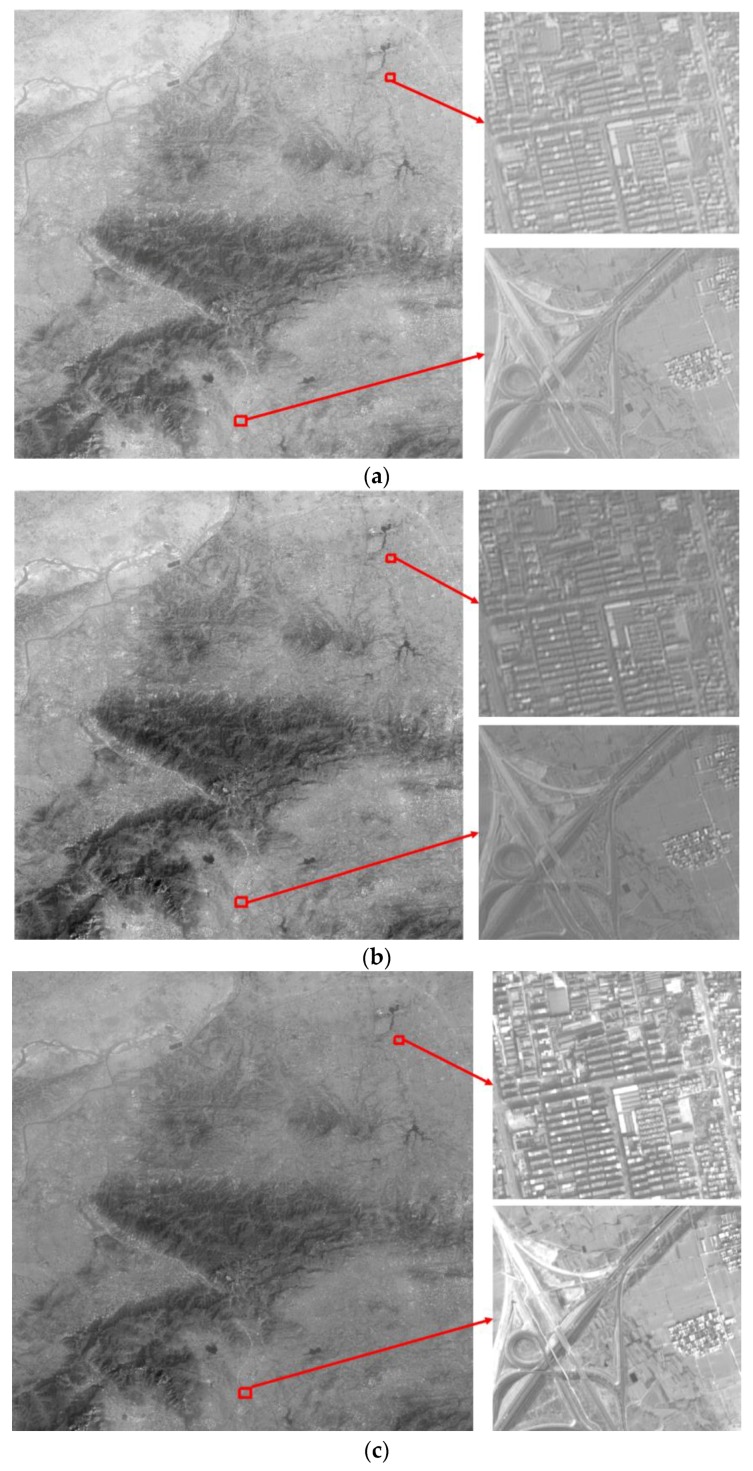
Comparison of the original image and the final result: (**a**) original forward image and its local amplification (**b**) original backward image and its local amplification (**c**) the final result and its local amplification.

In summary, the resolution of original ZY-3 forward and backward images shown in Figure 15a,b is enhanced by the blind SR reconstruction algorithm. Then, the difference in brightness and contrast among sub-block SR reconstructed images is eliminated using the improved Wallis dodging algorithm. Finally, a high-resolution image mosaicking without a seam line is obtained, as shown in Figure 15c. As we can see from Figure 15, the building and road in the two original images are fuzzy, while the edges of the building and road in the final image are clear and distinct, with more details.

## 5. Conclusions 

The Wallis dodging algorithm can remove the non-homogeneity of the brightness and contrast of multiple images. However, the existing algorithms cannot be applied to large-scale SR images. An efficient, improved weighted Wallis algorithm is proposed in this study. This algorithm uses a novel sequence of adjustment to avoid spatial transfer and accumulation of errors. A total of 900 SR images have been successfully tested by this method. The proposed method can effectively adjust the brightness and contrast differences among large-scale SR images and achieve a seamless image mosaicking with good visual effects compared with four other methods. This method is also suitable for color balance and storage of a large-scale image database.

In the experiment, large-scale waters and mists have a significant influence on the results and should be removed in advance. Therefore, investigating and exploring how to detect these special areas efficiently and accurately are necessary. An objective and effective assessment method should also be presented. This method is able to evaluate the precision and accuracy of our algorithm effectively.

## Figures and Tables

**Table 1:** The nominal values of metric Q of two reconstructed images.

Image Reconstructed by Different Algorithms	The Value of *Q*
The blind SR reconstruction algorithm	20.6348
The bicubic interpolation algorithm	13.0267

**Table 2:** The quality evaluation of images processed by different algorithms.

Different Image	Average Gradient	Information Entropy	PSNR
Original right image	3.0945	5.8662	
Right image after dodging based on histogram matching	7.252	5.5439	42.8714
Right image after dodging based on information entropy	2.8257	5.7266	43.0832
Right image after dodging based on Wallis filter	3.9136	5.8812	67.2056
